# BARRIERS AND FACILITATORS IN USING THE E-PAINSUPPORT INTERVENTION FOR ADRD PATIENTS AND CAREGIVERS

**DOI:** 10.1093/geroni/igad104.1125

**Published:** 2023-12-21

**Authors:** Masako Mayahara

**Affiliations:** Washington University in St. Louis, St. Louis, Missouri, United States

## Abstract

Pain is one of the most common comorbidities of ADRD and is experienced by 45.8% of ADRD patients. The major barrier to effective pain management is poor adherence to pain management regimens, due in part to caregiver lack of knowledge and lack of self-efficacy in administering analgesics. Pain in ADRD patients is associated with depression, wandering, agitation, and aggression. Digital applications may facilitate pain management by: (1) delivering education to increase knowledge and self-efficacy, (2) expediting pain reporting to nurses, and (3) improving adherence to pain management protocols. e-PainSupport is a self-administered, digital pain management application. It has three elements: (a) Educational Module, (b) Patient Pain Record, and (c) Pain Summary for Nurses. The efficacy of the app is currently being tested in a randomized controlled trial funded by the National Institutes of Health. The preliminary findings support the efficacy of e-PainSupport in reducing patient pain intensity and increasing caregivers’ self-efficacy and knowledge. However, the current intervention relies on patients’ self-reporting of pain and thus is not appropriate for all ADRD patients. Therefore, the purpose of this study is to explore facilitators for and barriers to using e-PainSupport for ADRD patients and caregivers. The data will be collected through post-intervention, semi-structured interviews of nurses and caregivers with patients in the e-PainSupport condition. Findings will inform future adaptations of e-PainSuport for ADRD patients and caregivers.

